# *DoMYB75* coordinately regulates polysaccharide and anthocyanin biosynthesis in *Dendrobium officinale*

**DOI:** 10.1093/hr/uhaf291

**Published:** 2025-10-25

**Authors:** Chunmei He, Danqi Zeng, Mingze Zhang, Can Si, Shoujie Li, Jing Chen, Hongyu Shi, Guangyi Dai, Zhong-Jian Liu, Jun Duan

**Affiliations:** Guangdong Provincial Key Laboratory of Applied Botany, State Key Laboratory of Plant Diversity and Specialty Crops, Key Laboratory of National Forestry and Grassland Administration on Plant Conservation and Utilization in Southern China, South China Botanical Garden, Chinese Academy of Sciences, Guangzhou 510650, China; College of Life Sciences, University of the Chinese Academy of Sciences, Beijing 100049, China; Guangdong Provincial Key Laboratory of Applied Botany, State Key Laboratory of Plant Diversity and Specialty Crops, Key Laboratory of National Forestry and Grassland Administration on Plant Conservation and Utilization in Southern China, South China Botanical Garden, Chinese Academy of Sciences, Guangzhou 510650, China; College of Life Sciences, University of the Chinese Academy of Sciences, Beijing 100049, China; Key Laboratory of National Forestry and Grassland Administration for Orchid Conservation and Utilization at College of Landscape Architecture and Art, Fujian Agriculture and Forestry University, Fuzhou 350002, China; The School of Life Science and Agriculture, Qiannan Normal University for Nationalities, Duyun 558000, China; Guangdong Provincial Key Laboratory of Applied Botany, State Key Laboratory of Plant Diversity and Specialty Crops, Key Laboratory of National Forestry and Grassland Administration on Plant Conservation and Utilization in Southern China, South China Botanical Garden, Chinese Academy of Sciences, Guangzhou 510650, China; Guangdong Provincial Key Laboratory of Applied Botany, State Key Laboratory of Plant Diversity and Specialty Crops, Key Laboratory of National Forestry and Grassland Administration on Plant Conservation and Utilization in Southern China, South China Botanical Garden, Chinese Academy of Sciences, Guangzhou 510650, China; Guangdong Provincial Key Laboratory of Applied Botany, State Key Laboratory of Plant Diversity and Specialty Crops, Key Laboratory of National Forestry and Grassland Administration on Plant Conservation and Utilization in Southern China, South China Botanical Garden, Chinese Academy of Sciences, Guangzhou 510650, China; College of Life Sciences, University of the Chinese Academy of Sciences, Beijing 100049, China; Guangdong Provincial Key Laboratory of Applied Botany, State Key Laboratory of Plant Diversity and Specialty Crops, Key Laboratory of National Forestry and Grassland Administration on Plant Conservation and Utilization in Southern China, South China Botanical Garden, Chinese Academy of Sciences, Guangzhou 510650, China; College of Life Sciences, University of the Chinese Academy of Sciences, Beijing 100049, China; Guangdong Provincial Key Laboratory of Applied Botany, State Key Laboratory of Plant Diversity and Specialty Crops, Key Laboratory of National Forestry and Grassland Administration on Plant Conservation and Utilization in Southern China, South China Botanical Garden, Chinese Academy of Sciences, Guangzhou 510650, China; College of Life Sciences, University of the Chinese Academy of Sciences, Beijing 100049, China; Key Laboratory of National Forestry and Grassland Administration for Orchid Conservation and Utilization at College of Landscape Architecture and Art, Fujian Agriculture and Forestry University, Fuzhou 350002, China; Guangdong Provincial Key Laboratory of Applied Botany, State Key Laboratory of Plant Diversity and Specialty Crops, Key Laboratory of National Forestry and Grassland Administration on Plant Conservation and Utilization in Southern China, South China Botanical Garden, Chinese Academy of Sciences, Guangzhou 510650, China; College of Life Sciences, University of the Chinese Academy of Sciences, Beijing 100049, China

## Abstract

*Dendrobium officinale*, a valuable medicinal plant, contains bioactive mannan polysaccharides that exert significant health-promoting effects in humans and serve as key quality markers for *D. officinale* products. However, the regulatory mechanisms underlying bioactive polysaccharide biosynthesis in plants remain poorly understood. In this study, we identified an anthocyanin-specific regulator, DoMYB75, as a key transcriptional activator of mannan polysaccharide biosynthesis in *D. officinale*. We demonstrated that DoMYB75 directly binds to the promoters of *CELLULOSE SYNTHASE-LIKE A* genes (*DoCSLA*s) and activate their expression. Genetic evidence showed that *DoMYB75* silencing reduced mannose and glucose content of water-soluble polysaccharides (WSPs) and downregulated *DoCSLA*s expression, whereas *DoMYB75* overexpression significantly increased these monosaccharide levels and upregulated *DoCSLA*s expression. Interestingly, *Ubi:DoMYB75* transgenic transformants exhibited enhanced anthocyanin accumulation. Further investigation revealed that DoMYB75 promotes anthocyanin biosynthesis by directly binding to and activating the *DoANS* promoter. Additionally, *DoMYB75* overexpression markedly improved total antioxidant capacity and drought tolerance. Our findings provide novel insights into the dual regulatory role of MYB transcription factors in coordinating polysaccharide and anthocyanin biosynthesis, as well as the adaptive mechanisms of *Dendrobium* orchids under drought stress.

## Introduction

*Dendrobium*, one of the largest and most economically important genera in the Orchidaceae family, includes *Dendrobium officinale* (also known as *Dendrobium catenatum*), a widely consumed species valued for its health-promoting properties. The primary bioactive components of *D. officinale* are its polysaccharides, which exhibit potent radical scavenging and immunomodulatory activities [1–3]. A recent study further suggests that these polysaccharides promote a healthy gut microbiota and enhance host immunity [4].

Increasing studies demonstrate that mannan is the main polysaccharide of *D*. *officinale* [1, 5, 6]. These polysaccharides play vital roles in energy storage, structural integrity, and stress tolerance, contributing to *D. officinale'*s survival in natural habitats. Mannan polysaccharides characterized by a backbone of β-1,4–linked mannosyl residues [7]. These polysaccharides display remarkable structural diversity and abundance variations across species, tissues, and developmental stages. Although they predominantly serve as structural components of cell walls in most plants, certain species have evolved specialized mechanisms for mannan storage. Notable examples include the seed endosperm of *Mauritia flexuosa* [8], stems of *Dendrobium* species [9], and corms of *Amorphophallus konjac* [10], where substantial mannan accumulates in dedicated storage organelles rather than cell walls.

Polysaccharide biosynthesis occurs through four key stages: nucleotide sugar donor activation, initiation, elongation, and termination [11]. Like most hemicelluloses, mannan biosynthesis takes place in the Golgi apparatus using activated sugar donors (nucleotide sugar) [12]. Research has shown that cellulose synthase-like A (CSLA) enzymes can synthesize β-linked glucomannan from GDP-mannose and GDP-glucose, and produce pure β-linked mannan when only GDP-mannose is available [13, 14], indicating their crucial role in forming β-1,4–linked mannosyl backbones. Functional studies have demonstrated that *Arabidopsis thaliana* CSLA proteins (AtCSLA2, AtCSLA3, AtCSLA7, and AtCSLA9) primarily synthesize glucomannan, a mannan polysaccharide [15]. In contrast, TaCSLA12 from wheat (*Triticum aestivum*), which shows preferential expression in developing endosperm, is responsible for the production of β-1,4-mannan, another type of mannan polysaccharide [16]. Our previous work identified eight *CSLA* genes in *D*. *officinale*, with *DoCSLA6* specifically implicated in mannan polysaccharide biosynthesis [9, 17]. These collective findings underscore the conserved function of *CSLA* genes in mediating β-1,4-linked mannan production across plant species.

Despite this progress, the regulatory mechanisms controlling mannan biosynthesis remain poorly understood. R2R3-MYB transcription factors represent promising candidates for such regulation, given their well-established roles in diverse biological processes including secondary cell wall biosynthesis [18–21]. As hemicellulose represents a major polysaccharide component of secondary cell walls, with mannan polysaccharide constituting a significant hemicellulose subtype [22, 23], investigating MYB TF involvement could reveal important regulatory networks. Although MYB TFs have been characterized in orchids [24, 25], current research has focused primarily on their roles in anthocyanin biosynthesis and development [26–31], leaving their potential regulation of polysaccharide biosynthesis largely unexplored.

In this study, we identified and functionally characterized DoMYB75, a subgroup 6 member in *D*. *officinale*, revealing its novel dual role in both mannan biosynthesis and anthocyanin accumulation. Furthermore, we demonstrated DoMYB75's involvement in drought stress response. Our findings provide mechanistic insights into the coordinated regulation of polysaccharide and secondary metabolite biosynthesis, while also identifying promising molecular targets for genetic improvement of *Dendrobium* cultivars.

## Results

### The *D. officinale* with purple stem contains higher levels of polysaccharides

**Figure 1:**
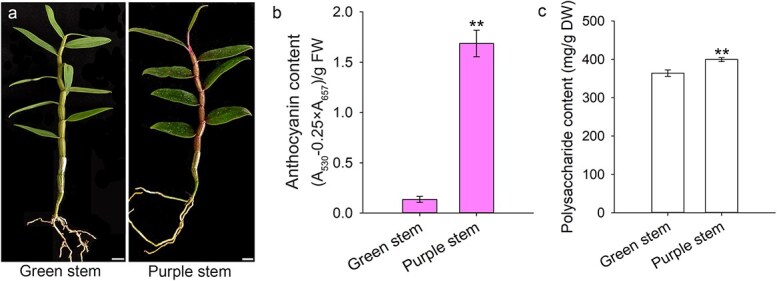
Comparative analysis of anthocyanins and water-soluble polysaccharides (WSPs) in *D*. *officinale* cultivars. (a) Phenotypic distinction between green-stem and purple-stem cultivars of *D*. *officinale* (scale bar = 1 cm). (b) Quantitative comparison of anthocyanin content. FW, fresh weight. (c) Quantitative comparison of WSP content. DW, dry weight. Data in (b) and (c) present mean ± SD (standard deviation) of three biological replicates. Double asterisks indicate significant differences (Student's *t*-test, *P* < 0.01).

During cultivation of *D*. *officinale*, we observed distinct phenotypic variations among cultivars, particularly in stem coloration. While some cultivars exhibited green stems, others displayed purple stems with vivid reddish-purple spotting (Fig. 1a). The concentration of anthocyanin in purple stems was markedly elevated, being approximately 12 times greater than that in green stems (Fig. 1b). This purple-stem phenotype was associated with significantly higher water-soluble polysaccharide (WSP) content compared to green-stem cultivars. Quantitative analysis revealed WSP levels of 399.69 mg/g dry weight (DW) in purple-stem cultivars versus 364.15 mg/g DW in green-stem cultivars (Fig. 1c), representing a 9.8% increase in polysaccharide content. This correlation between stem pigmentation and polysaccharide accumulation suggests a potential link between anthocyanin biosynthesis and carbohydrate metabolism in *D*. *officinale*.

### Expression analysis of *DoMYB75* from *D*. *officinale*

**Figure 2:**
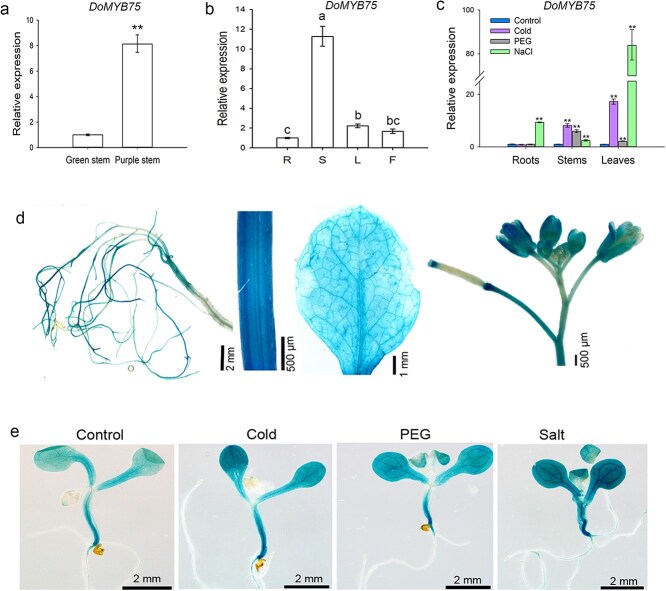
Expression profiling of *DoMYB75* gene. (a) Comparative transcript levels of *DoMYB75* between green-stem and purple-stem cultivars. (b) Tissue-specific expression patterns of *DoMYB75* in roots (R), stems (S), leaves (L), and flowers (F). (c) Expression dynamics of *DoMYB75* under abiotic stress treatments: cold (4°C), osmotic stress (15% PEG), and salinity (250 mM NaCl). (d) Histochemical determination of GUS activity in root, stem, leaf and flower of *proDoMYB75*:*GUS* transgenic *Arabidopsis* tissues. (e) GUS staining shows the effect of cold (4°C), 15% PEG and salinity (150 mM NaCl) treatments on the expression of *proDoMYB75*:*GUS* fusion in transgenic *Arabidopsis*. Data in (a-c) represent means ± SD of three biological replicates. Double asterisks in (a) and (c) indicate significantly different values (Student's *t*-test, *P* < 0.01). Significance analysis in Fig. 2c was conducted separately for each organ. Lowercase letters in (b) indicate significantly different values (Duncan's multiple range test, *P* < 0.05).

The expression of the R2R3-MYB transcription factor *DoMYB75* was found to correlate with WSP biosynthesis in *D*. *officinale* [24]. Comparative analysis revealed significantly higher *DoMYB75* expression levels in purple-stem cultivars compared to their green-stem counterparts (Fig. 2a), consistent with the observed differences in WSP content. Given that stems serve as the primary storage organ for polysaccharides in *D*. *officinale*, we performed comprehensive expression profiling across various tissues. Quantitative RT-PCR (qRT-PCR) analysis demonstrated ubiquitous *DoMYB75* expression, with predominant accumulation in stems (Fig. 2b).

Notably, *DoMYB75* exhibited transcriptional induction in response to abiotic stresses (cold, drought, and salinity) in both stems and leaves (Fig. 2c), mirroring the stress-responsive expression patterns previously reported for key mannan biosynthetic genes (*DoPMM* and *DoCSLAs*) [17, 32]. To further characterize *DoMYB75* expression, we conducted promoter-GUS fusion assays, which revealed β-glucuronidase activity in all examined organs (roots, stems, leaves, and flowers), with particularly intense staining in stems (Fig. 2d) - a finding that corroborated our qRT-PCR results. The enhancement of GUS staining in both stems and cotyledons was observed under cold (4°C), 15% (w/v) PEG, and salinity (150 mM NaCl) treatments (Fig. 2e). These results were in agreement with the high expression level of *DoMYB75* in stems displayed by qRT-PCR. These collective findings demonstrate that *DoMYB75* exhibits coordinated expression patterns with mannan biosynthetic genes, both spatially and in response to environmental cues, suggesting a functional relationship between this transcription factor and polysaccharide metabolism in *D*. *officinale*.

### Characterization of DoMYB75 protein

**Figure 3:**
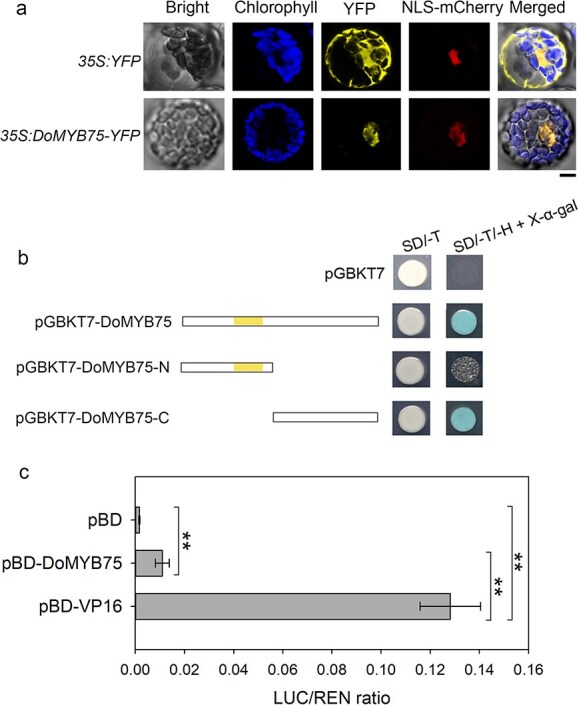
Functional characterization of transcription factor DoMYB75. (a) Subcellular localization of DoMYB75. The DoMYB75-YFP fusion protein was transiently expressed in *Arabidopsis* mesophyll protoplasts, showing nuclear localization as evidenced by co-localization with the nuclear marker NLS-mCherry (scale bar = 5 μm). (b) Transcriptional activation of DoMYB75 in yeast cells. DoMYB75-N and DoMYB75-C indicated the N-terminal (1–134 aa) and C-terminal (135–290 aa) of DoMYB75, respectively. SD/-T, SD-Trp medium; SD/-T/-H + X-*α*-gal, SD-Trp-His medium supplemented with X-*α*-gal. The yellow rectangle represents the MYB-like DNA-binding domain. (c) Transcriptional activation of DoMYB75 in *Nicotiana benthamiana* leaves. Values are means ± SD of six independent biological replicates. Double asterisks indicate significantly different values (Student's *t*-test, *P* < 0.05). pBD (empty vector) and pBD-VP16 (VP16 activation domain fusion) served as negative and positive controls, respectively.

Phylogenetic analysis assigned DoMYB75 to the R2R3-MYB subgroup 6 clade (Fig. S1). Its protein sequence possessed the conserved R2 and R3 MYB DNA-binding domains (Fig. S2). Notably, sequence alignment revealed limited similarity between DoMYB75 and *Arabidopsis* AtMYB75 (Fig. S2). To determine the subcellular localization of DoMYB75, we performed transient expression of *35S*:*DoMYB75*-*YFP* fusion construct in *Arabidopsis* mesophyll protoplasts. Confocal microscopy revealed co-localization with the nuclear marker NLS-mCherry, confirming exclusive nuclear accumulation of DoMYB75 (Fig. 3a), which aligns with its predicted role as a DNA-binding transcription factor. To investigate the transactivation activity of DoMYB75, we fused the full-length sequence and its deletion derivatives [DoMYB75-N (1–134 amino acids, aa) and DoMYB75-C (135–290 aa)] with GAL4 DNA-binding domain (GAL4-DB), and expressed them in yeast strain AH109. As shown in Fig. 3b, the yeast cells harboring the negative control pGBKT7 or pGBKT7-DoMYB75-N plasmids did not complement the histidine deficiency, while the yeast cells carrying pGBKT7-DoMYB75 or pGBKT7-DoMYB75-C plasmids grew well on synthetic medium lacking histidine and showed X-*α*-galactosidase activity. These results indicated that DoMYB75 acts as a transcriptional activator and the C-terminal portion of DoMYB75 is responsible for the transactivation activity in yeast. To further confirm the transcriptional activation activity of DoMYB75, we performed a dual-luciferase reporter (DLR) assay using transient expression in *Nicotiana benthamiana* leaves. Our results showed that the effectors of pBD-DoMYB75 and pBD-VP16 (positive control) significantly promoted the expression of the reporter gene *LUC* compared with negative control pBD (Fig. 3c). These results indicated that DoMYB75 encodes a nuclear protein with transcriptional activation activity.

### DoMYB75 directly binds to the promoters of *DoCSLAs in vitro* and activates their expression

**Figure 4:**
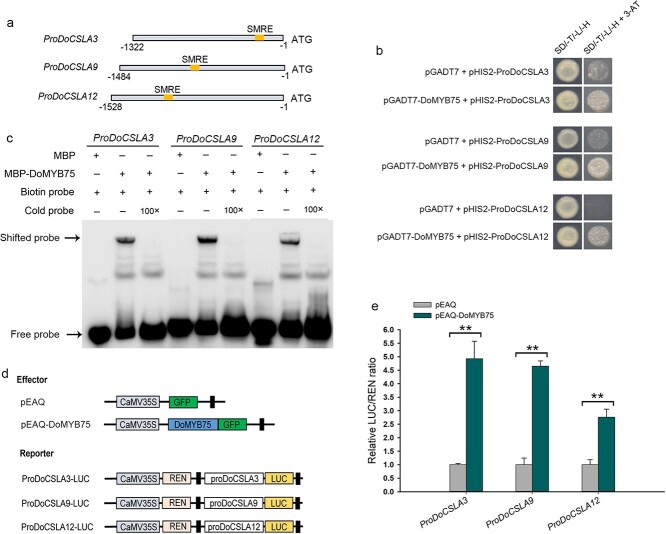
Analysis of interactions between DoMYB75 and *DoCSLA* promoters. (a) Promoter sequence analysis revealed the presence of a single SMRE (secondary wall MYB-responsive element) in *DoCSLA3*, *DoCSLA9*, and *DoCSLA12* promoters. (b) Yeast one-hybrid (Y1H) assays showed the binding activity of DoMYB75 protein with promoters of *DoCSLA3*, *DoCSLA9* and *DoCSLA12*. SD/-T/-L/-H, SD-Trp-Leu-His medium. SD/-T/-L/-H + 3-AT, SD-Trp-Leu-His medium supplemented with 120 mM 3-amino-1,2,4- triazole (3-AT). (c) Electrophoretic mobility shift assays (EMSA) confirmed the direct binding of DoMYB75 protein to *DoCSLA3*, *DoCSLA9*, and *DoCSLA12* promoters *in vitro*. (d) Schematic diagram of the effector and reporters used in Dual luciferase reporter (DLR) assays. (e) DoMYB75 activated the promoters of *DoCSLA3*, *DoCSLA9* and *DoCSLA12* in *N*. *benthamiana* leaves. Values represent mean ± SD of six independent biological replicates. Double asterisks indicate statistically significant differences compared to the control by Student's *t*-test (*P* < 0.01).

Given the expression pattern of *DoMYB75*, we hypothesized that *DoMYB75* played a crucial role in the biosynthesis of polysaccharides in *D*. *officinale*. Numerous MYB transcription factors directly bind to the secondary wall MYB-responsive element (SMRE) consensus sequences ACC(A/T)A(A/C)(T/C), and activate the expression of secondary wall biosynthetic genes [33, 34]. We conducted an analysis of *cis*-acting elements within the promoters of genes involved in the polysaccharide biosynthesis. Our findings revealed that three *CSLA* genes (*DoCSLA3*, *DoCSLA9*, and *DoCSLA12*) contain a single SMRE within their respective promoters (Fig. 4a). We conducted yeast one-hybrid (Y1H) analysis to ascertain whether DoMYB75 targets *DoCSLAs* directly. The Y1H results showed that the growth of yeast cells, which contained pGADT7 and the promoter of *DoCSLA3*/*-9*/*-12* was inhibited on synthetic dropout (SD)/-T/-L/-H medium with 120 mM 3-amino-1,2,4-triazole (3-AT) (Fig. 4b). In contrast, the yeast cells containing pGADT7-DoMYB75 and promoter of *DoCSLA3*/*-9*/*-12* could grow well on SD/-T/-L/-H medium with 120 mM 3-AT (Fig. 4b). These results suggest that DoMYB75 can bind to the promoters of *DoCSLAs* in yeast. Then, we conducted Electrophoretic Mobility Shift Assay (EMSA) to confirm the binding activity *in vitro*. We used the promoter fragments of *DoCSLAs* containing the SMRE site as probes to test the interactions. Our results showed that the maltose binding protein (MBP)-DoMYB75 recombinant protein can directly bind to these labeled probes derived from *DoCSLA* promoters (Fig. 4c). The shifted bands could be competed out when supplemented with unlabeled probes (cold probes) as competitors (Fig. 4c). In contrast, the MBP protein could not bind to these labeled probes and only showed free probes (Fig. 4c).

To determine whether DoMYB75 activates the *DoCSLA* promoters, we performed DLR experiments to verify the association between DoMYB75 and *DoCSLA* promoters. The full length of *DoMYB75* was cloned into the pEAQ vector to produce effector, and the *DoCSLA* promoters were separately fused to the firefly luciferase (*LUC*) gene to generate the reporters (Fig. 4d). The DLR assays showed that in the presence of DoMYB75, the promoter activities of *DoCSLAs* significantly increased (Fig. 4e). These findings collectively support that DoMYB75 directly binds to the promoters of *DoCSLAs* and activates their expression. This suggests that DoMYB75 may function as a transcriptional activator for the biosynthesis of mannans in *D*. *officinale*.

### DoMYB75 is pivotal in mannan biosynthesis in *D*. *officinale*

**Figure 5:**
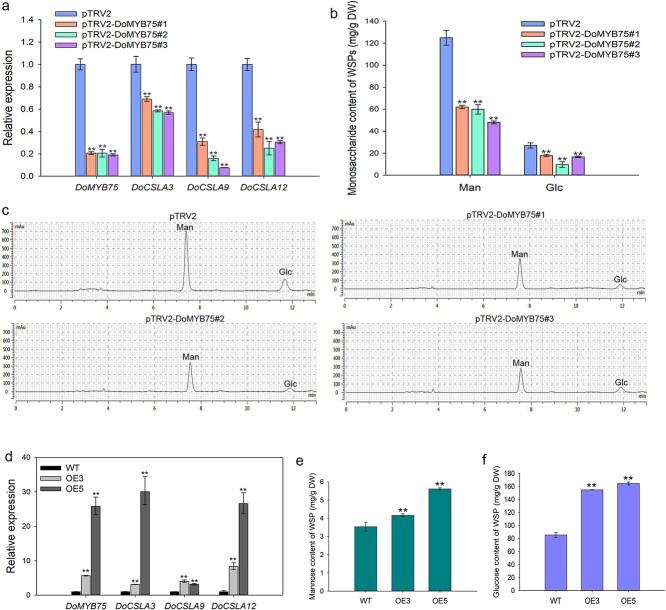
*DoMYB75* regulates mannan biosynthesis in *D*. *offcinale*. (a) Relative expression levels of *DoMYB75* and its target genes (*DoCSLA3*, *DoCSLA9*, and *DoCSLA12*) in control (pTRV2) and *DoMYB75*-silenced plants. Control, pTRV2; pTRV2-DoMYB75, *DoMYB75*-silenced *Dendrobium*. (b) Monosaccharide composition analysis of water-soluble polysaccharides (WSPs) from control (pTRV2) and *DoMYB75*-silenced plants by high-performance liquid chromatography (HPLC). Man, mannose; Glc, glucose. Stem tissues were used for analysis. Control, pTRV2; pTRV2-DoMYB75, *DoMYB75*-silenced *Dendrobium*. (c) HPLC chromatogram of monosaccharide composition analysis. Man, mannose; Glc, glucose. Negative control, pTRV2; pTRV2-DoMYB75, *DoMYB75*-silenced *Dendrobium*. (d) Overexpression of *DoMYB75* significantly up-regulated the expression of *DoMYB75*, *DoCSLA3*, *DoCSLA9* and *DoCSLA12*. (e) and (f) *DoMYB75* overexpression increased mannose and glucose content in protocorm-like bodies (PLBs). PLBs from wild-type (WT) and *DoMYB75*-overexpressing transformants (OE3 and OE5) were analyzed. Values in (a), (b) and (d-f) represent the mean ± SD of three biological experiments. Statistical significance is indicated by asterisks: ^*^P < 0.05 and ^**^P < 0.01 as determined by one-way ANOVA followed by Dunnett's test. WT, wild-type; Two *Ubi*:*DoMYB75* transgenic transformants: OE3 and OE5.

To investigate the function of DoMYB75 in *D*. *officinale* polysaccharide biosynthesis, we conducted a loss-of-function analysis using virus-induced gene silencing (VIGS) on *DoMYB75*. Our qRT-PCR results revealed a significant decrease in the transcript levels of *DoMYB75* across three silenced *Dendrobium* plants compared to the control plants (Fig. 5a). This reduction was also observed in its target genes, *DoCSLA3*, *DoCSLA9*, and *DoCSLA12* (Fig. 5a). We then extracted water-soluble polysaccharides from both the control and *DoMYB75*-silenced plants and quantified their monosaccharide composition using high-performance liquid chromatography (HPLC). The findings indicated that mannose and glucose levels were significantly reduced in the *DoMYB75*-silenced plants when compared to the control (Fig. 5b and c). Specifically, the mannose content in the control was approximately 124.94 mg/g DW, whereas none of the DoMYB75-silenced *D. officinale* plants had a mannose content exceeding 65.00 mg/g DW (Fig. 5b and c).

To provide robust evidence regarding the role of *DoMYB75* in mannan biosynthesis, we overexpressed *DoMYB75* in *D*. *officinale.* We generated a total of 14 independent transgenic transformants, and chose two transformants (OE3 and OE5) randomly for further analysis. Compared with the wild-type (WT), the expression of *DoMYB75* in these two transgenic transformants increased by at least 5-fold (Fig. 5d). *DoCSLAs* were significantly upregulated in two transgenic transformants (Fig. 5d), indicating that DoMYB75 triggers the expression of *DoCSLAs* and may lead to the accumulation of mannan in *D*. *officinale*. WSPs were extracted from both transgenic protocorm-like-bodies (PLBs) and WT PLBs, and used for monosaccharide composition analysis. The mannose content in the WT was found to be 3.54 mg/g DW, whereas it was observed to be 4.17 and 5.615 mg/g DW in the two transgenic transformants, respectively (Fig. 5e). Additionally, the glucose content in the WT was 85.37 mg/g DW, while both transgenic transformants exhibited a glucose content exceeding 150 mg/g DW (Fig. 5f). These findings suggest that DoMYB75 plays an essential role in *D*. *officinale* mannan biosynthesis.

### DoMYB75 regulates anthocyanin biosynthesis in *D*. *officinale*

**Figure 6:**
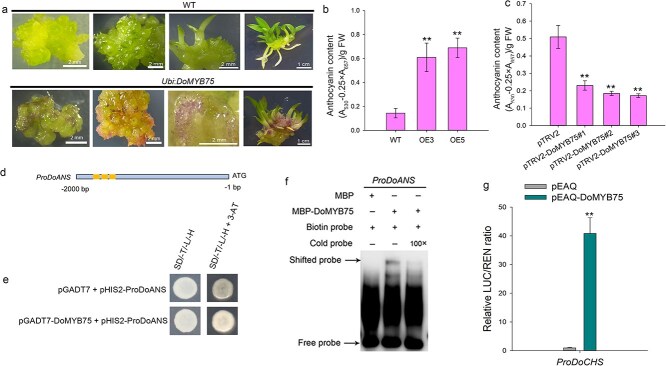
*DoMYB75* regulates anthocyanin accumulation in *D*. *officinale*. (a) Phenotypic observation showing increased anthocyanin pigmentation in *Ubi:DoMYB75* transgenic transformants compared to wild-type (WT) plants. (b) Quantitative analysis of anthocyanin content in WT and two independent *Ubi:DoMYB75* transgenic transformants (OE3 and OE5). Data represent mean ± SD of three biological experiments. Double asterisks indicate statistically significant differences between WT and transgenic transformants (one-way ANOVA followed by Dunnett's test, *P* < 0.01). WT, wild-type; Two *Ubi*:*DoMYB75* transgenic transformants: OE3 and OE5. (c) Analysis of anthocyanin content in control (pTRV2) and silencing *DoMYB75 Dendrobium* plants (pTRV2-DoMYB75). Data represent mean ± SD of three biological experiments. Double asterisks indicate statistically significant differences between control and silencing *DoMYB75* (one-way ANOVA followed by Dunnett's test, *P* < 0.01). (d) The locations of three SMREs within the *DoANS* promoter region are illustrated. (e) Yeast one-hybrid (Y1H) assays demonstrating DoMYB75 binding to the *DoANS* promoter. SD/-T/-L/-H, SD-Trp-Leu-His medium. SD/-T/-L/-H + 3-AT, SD-Trp-Leu-His medium supplemented with 80 mM 3-AT. (f) Electrophoretic mobility shift assay (EMSA) confirming *in vitro* binding of recombinant DoMYB75 protein to the *DoANS* promoter. (g) DoMYB75 activated the promoter of *DoANS* in *N*. *benthamiana* leaves. Data are shown as means ± SD of six independent assays. Double asterisks indicate statistically significant differences by Student's *t*-test (*P* < 0.01).

Intriguingly, the PLBs, leaves and shoots of *DoMYB75* overexpressing transformants exhibited a purple-pigmentation phenotype (Fig. 6a), indicating that overexpression of *DoMYB75* leads to anthocyanin accumulation in *D*. *officinale*. In contrast, the WT plants cultured under the same condition did not show any visible purple pigments (Fig. 6a). The anthocyanin content exhibited a significant increase in the two transgenic transformants compared to the WT (Fig. 6b). Silencing *DoMYB75* resulted in significantly lower anthocyanin content (Fig. 6c). To explore the regulatory role of DoMYB75 on anthocyanin biosynthesis, we isolated the promoters of key genes in this pathway, including chalcone synthase gene *DoCHS*, chalcone isomerase gene (*DoCHI*) and anthocyanidin synthase gene (*DoANS*) (Text S1). Subsequently, these were used for the prediction of *cis*-acting elements. Three SMRE sites were identified within the promoter region of the *DoANS* (Fig. 6d), which encodes the enzyme that catalyzes the penultimate step in the biosynthesis of anthocyanins [35]. To determine whether DoMYB75 binds to the *DoANS* promoter, the promoter fragment of *DoANS* containing the SMRE sites was cloned into pHIS2 to generate pHIS2-ProDoANS construct. The pGADT7-DoMYB75 plasmid and pHIS2-ProDoANS plasmid were co-transformed into the Y187 yeast strain for Y1H assays. Yeast cells containing pGADT7-DoMYB75 and pHIS2-ProDoANS grew better than the negative control (co-transformed with pGADT7 and pHIS2-ProDoANS) on SD/-T/L/-H medium containing 3-AT, indicating that DoMYB75 could directly bind to the promoter region of *DoANS* and activate the expression of reporter gene in yeast (Fig. 6e). Subsequently, we conducted EMSA to evaluate the interaction between DoMYB75 and the *DoANS* promoter. *DoANS* promoter fragment containing the SMRE sites (located at −1616 to −1674 bp) were labeled with biotin and used as probes for EMSA analysis. The recombinant MBP-DoMYB75 protein directly interacted with the biotin probe, which was derived from the *DoANS* promoter, leading to observed mobility shifts (Fig. 6f). The retarded bands could be competed out when supplemented with unlabeled competitive probe (cold probe) (Fig. 6f). To validate the transcriptional activator role of DoMYB75 on *DoANS* expression, we employed a DLR assay. Our findings revealed that the promoter activity of *DoANS* was markedly elevated in the presence of DoMYB75 relative to the control (Fig. 6g). These results suggest that DoMYB75 regulates the expression of *DoANS* by directly binding to the *DoANS* promoter, thereby modulating the biosynthesis of anthocyanins in *D*. *officinale*.

### DoMYB75 plays a role in drought tolerance

**Figure 7:**
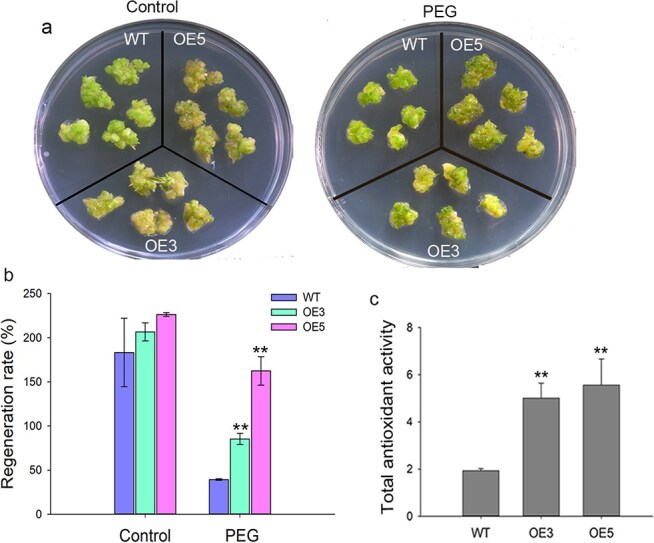
Overexpression of *DoMYB75* increased PLB regeneration under drought stress in *D*. *officinale*. (a) and (b) Overexpression of *DoMYB75* facilitates PLB regeneration under 15% polyethylene glycol 6000 (PEG) treatment. The data represent the mean ± SD of six independent biological replicates. (c) Total antioxidant activity in WT and *Ubi*:*DoMYB75* transgenic transformants under control conditions. The data represent the mean ± SD of three biological replicates. ** indicates statistically significant differences between the WT and transgenic transformants by Student's *t*-test (*P* < 0.01). WT, wild-type; Two *Ubi*:*DoMYB75* transgenic transformants: OE3 and OE5.

*D*. *officinale* is a typical epiphytic orchid, which grows on the trunks of primary forests or rocks in mountains, and such living environment easily leads to water deficiency. To test the effect of DoMYB75 on the drought tolerance of *D*. *officinale*, the transgenic PLBs were treated with 15% PEG. Our results showed that the regeneration rate of transgenic transformants was not significantly different from that of WT under control (Fig. 7a and b). By contrast, the growth of WT and transgenic PLBs was repressed under PEG treatment, and the regeneration rate of transgenic transformants was significantly higher than that of WT (Fig. 7a and b). Following a 30-day cultivation period, the average regeneration rate of WT under PEG treatment was merely 39.36% (Fig. 7b). In contrast, transgenic PLBs demonstrated significantly higher regeneration rates than WT, registering at 85.35% and 162.46%, respectively (Fig. 7b). Given the robust antioxidant capacity of both polysaccharides and anthocyanins, we hypothesized that DoMYB75 can enhance the total antioxidant activity of *D*. *officinale*. The total antioxidant activity exhibited a significant increase in the two transgenic transformants compared to the WT (Fig. 7c). In the 3,3-diaminobezidine (DAB) experiment, PEG-exposed PLBs of WT exhibited a significantly larger area stained darkly brown compared to transgenic PLBs (Fig. S3). The findings suggest that overexpression of *DoMYB75* enhances plant capacity to eliminate H_2_O_2_ and diminishes its toxicity to reactive oxygen species. Consequently, this leads to an improvement in plant tolerance to drought stress.

## Discussion

**Figure 8:**
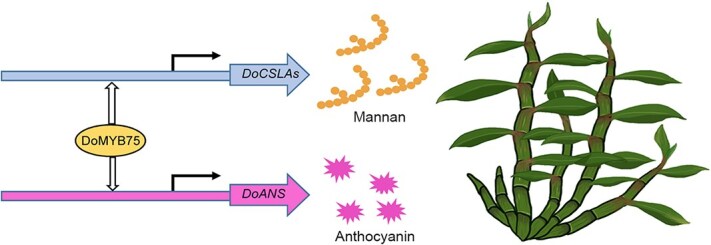
A proposed regulatory model of DoMYB75 in *D*. *officinale*. DoMYB75 simultaneously regulates polysaccharide and anthocyanin biosynthesis in *Dendrobium* orchids.

This study elucidates the dual regulatory role of DoMYB75 in coordinating the biosynthesis of polysaccharides and anthocyanins in *D*. *officinale*. We demonstrate that DoMYB75 directly binds to and activates the promoters of key biosynthetic genes (*DoCSLA*s and *DoANS*), thereby promoting the accumulation of both mannans and anthocyanins. Functional analyses revealed that *DoMYB75* overexpression not only enhances the production of these valuable metabolites but also significantly improves drought tolerance in *D*. *officinale*. Based on our findings, we propose a regulatory model wherein DoMYB75 serves as a master coordinator that simultaneously activates mannan biosynthesis (through *DoCSLA*s regulation) and anthocyanin production (*via DoANS* activation) (Fig. 8). These results provide the molecular evidence for MYB-mediated regulation of polysaccharide biosynthesis in medicinal orchids, addressing a critical knowledge gap in our understanding of bioactive compound production in this economically important plant family.

The R2R3-MYB family characterized by two MYB repeats (R2 and R3) that share high similarity with the corresponding domains in c-MYB [36]. Our sequence analysis confirmed that DoMYB75 contains these conserved R2 and R3 MYB repeats (Fig. S2), establishing its classification as a R2R3-MYB transcription factor. Extensive studies have demonstrated that R2R3-MYB proteins can function as either transcriptional activators or repressors in plants. For example, AtMYB75 from *Arabidopsis* acts as a transcriptional activator [37]. AtMYB17, whose C-terminal region exhibits strong activation activity in yeast [38]. MYB31, an R2R3-MYB protein in pepper, is located in the nucleus, and its C-terminal region acts as a transcriptional activator in yeast [39]. In addition to activators, R2R3-MYB proteins also act as repressors in plants [40–42]. *Arabidopsis* AtMYB4 acts as a transcriptional repressor and represses the promoter activities of *AtC4H*, *AtCHS*, *At4CL3* genes [43]. Most repressors belong to subgroup 4, which contains a bHLH-binding domain in the R3 repeat and two conserved motifs (C1 and C2 motifs) in the C-terminal region [44]. DoMYB75 belongs to the subgroup 6, and does not contain the bHLH-binding domain and two conserved protein motifs (Fig. S1), indicating that DoMYB75 is not a repressor. Transcriptional activity analysis showed that DoMYB75 functions as an activator in yeast and *N*. *benthamiana*, and its C-terminal region is responsible for the transcriptional activation. In addition, overexpression of *DoMYB75* in *D*. *officinale* showed that DoMYB75 plays a positive role in regulating the biosynthesis of polysaccharides and anthocyanins. Taken together, we present evidence that DoMYB75 is a transcriptional activator in plants.

*D*. *officinale* accumulates substantial quantities of bioactive polysaccharides, particularly glucomannan, which exhibits remarkable medicinal properties. Previous studies have established that CSLA family proteins mediate the biosynthesis of mannan polymers [45–47]. Currently available evidence indicates that only three transcription factors - AtMYB46, AtANAC041, and AtbZIP1 - have been reported to regulate *CSLA* gene expression [48]. To elucidate the regulatory network governing bioactive polysaccharide biosynthesis in *D. officinale*, we characterized a R2R3-MYB transcription factor, *DoMYB75*, whose expression profile positively correlates with polysaccharide accumulation in *D. officinale*. SMRE *cis*-element is one of the binding sequences of the R2R3-MYB protein. For example, a wheat (*Triticeae aestivum*) Myb10-D protein binds to the SMRE of 9-*cis*-epoxycarotenoid dioxygenase (*NCED*) gene promoter, and induces the expression of *NCED* in wheat [49]. Cotton (*Gossypium hirsutum*) GhMYB7 activates *GhCesA* expression through SMRE binding, promoting secondary wall cellulose deposition [50]. *Arabidopsis* AtMYB46 and AtMYB83 regulate secondary wall biosynthesis genes *via* SMRE-mediated transcriptional activation [34]. Our findings demonstrate that DoMYB75 positively regulates mannan biosynthesis through direct binding to *DoCSLA* promoters and transcriptional activation in *Dendrobium*. These results provide evidence that MYB transcription factors play conserved roles in regulating mannan biosynthesis across plant species.

Anthocyanins represent a major class of pigmentation compounds that contribute vivid coloration to plant organs, particularly flowers and fruits. It is noteworthy that anthocyanins also exert beneficial effects on human health. They exhibit antioxidant, free radical-scavenging, and anti-tumor capabilities, and can regulate multiple physiological processes such as lipid metabolism, inflammatory responses, and immune function [51]. The subgroup 6 of R2R3-MYB transcription factors been functionally characterized as key transcriptional activators of anthocyanin biosynthesis pathways [36]. In *Arabidopsis*, four well-characterized subgroup 6 members (AtMYB75, AtMYB90, AtMYB113, and AtMYB114) have been demonstrated to coordinately regulate anthocyanin production [52]. Phylogenetic analyses reveal that this regulatory function is evolutionarily conserved, with orthologous subgroup 6 R2R3-MYB TFs controlling anthocyanin biosynthesis in diverse species including *Cannabis sativa* L. [53], *Anthurium andraeanum* [54], and *Antirrhinum majus* [55]. In this study, DoMYB75 was found to effectively enhance anthocyanin accumulation in *D*. *officinale* (Fig. 6), indicating the conserved function of subgroup 6 R2R3-MYB TFs in anthocyanin biosynthesis. Studies have shown that MYB transcription factors regulate anthocyanin synthesis by regulating anthocyanin structural genes. PdMYB118 transcription has the ability to directly activate the promoters of *CHS1*, *DRF2*, and *ANS1* genes [56]. This activation is integral to the regulation of anthocyanin biosynthesis. PeMYBs could activate the expression of three downstream structural genes *F3H*, *DFR* and *ANS* in *Phalaenopsis* [27]. In addition to MYB transcription factors, other factors, such as bHLH, WRKY, and B-box proteins, have also been demonstrated to participate in the regulation of anthocyanin biosynthesis [57–59]. We presented experimental evidence in support that DoMYB75 is a direct regulator of *DoANS*. These findings are consistent with the established paradigm of subgroup 6 MYB TFs serving as positive regulators of anthocyanin biosynthesis across plant species.

Recent research has revealed that the bZIP TF ELONGATED HYPOCOTYL 5 (HY5) directly regulates both polysaccharide and anthocyanin biosynthesis in *D*. *officinale* by binding to the promoters of key biosynthetic genes (*DoGMPP2* and *DoPMT28* for polysaccharides; *DoF3H1* for anthocyanins) [60]. We demonstrated that DoMYB75 not only directly binds to the promoters of *DoCSLA*s to regulate the mannan biosynthesis but also mediates the anthocyanin accumulation through directly binding to the promoters of *DoANS*. This phenomenon of single transcription factors coordinating multiple metabolic pathways appears evolutionarily conserved, as evidenced by RcMYB1's simultaneous regulation of anthocyanin and carotenoid biosynthesis in roses [61]. Both *Dendrobium* polysaccharides and anthocyanins are well established as potent antioxidants. Overexpression of *DoMYB75* significantly boosts the total antioxidant activity and drought tolerance in *D*. *officinale*. However, our current study is yet to determine whether this enhanced drought tolerance is due to the polysaccharides of *D*. *officinale* or anthocyanins. A recent review has discussed the roles of flavonoids, in mitigating the effects of environmental stress in horticultural plants [62]. The limited and incomplete information provided still presents a significant challenge for us, necessitating further detailed investigation.

We identified a subgroup 6 R2R3-MYB transcription factor, DoMYB75, in *D*. *officinale*. The protein encoded by this gene was localized within the nucleus and exhibited self-activation activity in yeast. We successfully manipulated the expression of *DoMYB75* in *D*. *officinale* to investigate its function. Our findings verified that DoMYB75 regulated the expression of *DoCSLAs* by binding to their promoters, thereby controlling mannan accumulation. Furthermore, DoMYB75 influenced anthocyanin biosynthesis and enhanced drought tolerance. The R2R3-MYB transcription factor from subgroup 6 has been extensively recognized as a crucial upstream regulatory factor in the anthocyanin biosynthesis pathway. However, our study is the first to demonstrate that MYB genes from this subgroup are implicated in mannan biosynthesis. These findings offer a valuable genetic resource and theoretical guidance for crop breeding.

## Materials and methods

### Plant materials and treatments

Mature *Dendrobium officinale* Kimura et Migo plants were collected from *Dendrobium* nursery at South China Botanical Garden, Chinese Academy of Sciences (Guangzhou, Guangdong, China). The plant materials were authenticated by Professor Jun Duan. The roots, stems, leaves and flowers were collected from reproductive stage plants for gene expression analysis. Protocorm-like bodies (PLBs) and plantlets were grown in 1/2 Murashige and Skoog (MS) medium [63] in a growth chamber at a temperature of 24°C, 12-h photoperiod and irradiance of 40 μmol∙m^−2^∙s^−1^. For stress treatments, plantlets with a height of about 5 cm were subjected to cold (4°C), 15% polyethylene glycol 6000 (PEG-6000) and salinity (250 mM NaCl) treatments. After 24 hours of treatment, roots, stems, and leaves were separately harvested (6 plantlets per treatment), flash-frozen in liquid nitrogen, and isolated the total RNA. Each treatment included three independent biological replicates.

### Real time quantitative PCR (qRT-PCR)

Gene-specific qRT-PCR primers were designed using PrimerQuest™ Tool (Integrated DNA Technologies; https://www.idtdna.com/Primerquest), with all primer sequences listed in Table S1. qRT-PCR was performed on a LightCycler 480 System (Roche) following the manufacturer's protocol. Each reaction contained the Unique Aptamer™ qPCR SYBR® Green Master Mix (Novogene, Beijing) and was run for 40 cycles under the following conditions: 95°C denaturation for 15 s and 60°C annealing/extension for 1 min. Gene expression levels were normalized to the actin reference gene (NCBI accession: JX294908) and calculated using the 2^−ΔΔCt^ method [64].

### Transactivation activity analysis of DoMYB75

The full-length coding sequence (CDS) of *DoMYB75* and its truncated variants (DoMYB75-N, aa, 1–134; DoMYB75-C, aa, 135–290) were cloned into pGBKT7 (Clontech, USA) and transformed into the yeast strain AH109. Positive transformants were selected on SD/-Trp/-His medium containing X-*α*-Gal (5-Bromo-4-chloro-3-indolyl-*α*-D-galactoside) and incubated at 29°C for blue color development.

For in vivo transcriptional activity analysis, we employed a dual-luciferase transient expression system [65]. The CDS of *DoMYB75* was cloned into the pBD vector to generate DoMYB75-GAL4 fusion effector. The reporter system consisted of the firefly luciferase (LUC) gene under control of a minimal *CaMV 35S* promoter with five upstream GAL4-binding elements. *Agrobacterium tumefaciens* strain GV3101 harboring either the effector or reporter constructs were co-infiltrated into *Nicotiana benthamiana* leaves. Luciferase activities were measured 72 hours post-infiltration using the Dual-Luciferase® Reporter Assay System (Promega, Madison, WI, USA), with firefly luciferase values normalized to Renilla luciferase internal controls. A minimum of six independent biological replicates were performed for each experiment. All primers used in these analyses are listed in Table S1.

### Electrophoretic mobility shift assay (EMSA)

The MBP–DoMYB75 fusion protein expression vector was constructed and expressed in *Escherichia coli* BL21 (DE3) strain for MBP-DoMYB75 fusion protein production. The biotin-labeled DNA probes (promoter fragments of *DoCSLAs* and *DoANS* containing SMRE sequences) and non-biotin labelled (cold) competitor probes were synthesized for DNA-protein interaction analysis. EMSA was conducted using the LightShift™ Chemiluminescent EMSA Kit (Thermo Fisher Scientific, USA) following the manufacturer's instructions. The primers and DNA probes employed for EMSA are presented in Table S1.

### Transient expression assays

The full-length CDS of *DoMYB75* (excluding the stop codon) was cloned into the pEAQ vector to generate the effector construct. The promoter fragments of *DoCSLAs* and *DoANS* were individually inserted into the pGreenII 0800-LUC vector to create reporter constructs. *A. tumefaciens* GV3101 cells harboring the effector or reporter constructs were co-infiltrated into *N*. *benthamiana* leaves, followed by incubation under dark conditions at 25°C for 48 hours. After the dark treatment, the plants were transferred to controlled photoperiod conditions (16/8 h light/dark cycle with 100 μmol∙m^−2^∙s^−1^ light intensity) for 24 hours. The dual-luciferase reporter assay was then performed using the Dual-Luciferase® Reporter Assay System (Promega, Madison, WI, USA) in accordance with the manufacturer's protocol. A minimum of six biological replicates were performed for each experiment. All primer sequences are provided in Table S1.

### Virus-induced gene silencing (VIGS) assay

The *DoMYB75* fragment was cloned into the pTRV2 vector to generate the silencing recombinant vector (pTRV2-DoMYB75). *Agrobacterium*-mediated transformation facilitated the co-infiltration of pTRV1 and pTRV2-DoMYB75 into 1-year-old *D*. *officinale* plants, with co-infiltrated pTVR1 and pTRV2 plants serving as negative controls. Following infiltration, the plants were maintained under controlled conditions (12-h light, 500 μmol∙m^−2^∙s^−1^, 24°C). Leaf samples were collected 25 days post-infiltration for total RNA extraction and subsequent transcriptional analysis to assess silencing efficiency. At 45 days post-infiltration, the leaf samples were collected for anthocyanin analysis, and stem samples were harvested for polysaccharide analysis. The dried stems were pulverized into fine powder for water-soluble polysaccharide (WSP) extraction. The monosaccharide composition of WSPs was analyzed by high-performance liquid chromatography (HPLC) following derivatization. All primer sequences are provided in Table S1.

### PEG treatment and DAB staining

The protocorm-like bodies (PLBs) were sectioned into approximately 5 mm diameter segments and cultured on growth medium (½ MS basal salts supplemented with 2% (w/v) sucrose, 0.2 mg/l 1-naphthaleneacetic acid (NAA), 0.5 mg/l 6-benzylaminopurine (6-BA), and 0.6% (w/v) agar, pH 5.4) in a plant growth chamber for 10 days. Subsequently, the PLBs were transferred to fresh growth medium containing 15% (w/v) polyethylene glycol (PEG) for drought stress treatment. PLB explants cultured on PEG-free growth medium served as the control. After one month of culture, the regeneration rate for each genotype was calculated as previously described [66]. Regeneration rate (%) = (Fresh weight of PLBs after cultivation − Fresh weight of PLBs before cultivation) ÷ Fresh weight of PLBs before cultivation × 100%. PLB explants were collected and stained with 3,3′-diaminobenzidine (DAB) according to the instrument.

### Supporting information for methods

The methodological details, including vector construction and *Arabidopsis* transformation (Method S1), β-glucuronidase (GUS) staining (Method S2), overexpression vector construction and *Dendrobium officinale* transformation (Method S3), anthocyanin quantification (Method S4), monosaccharide composition analysis (Method S5), RNA extraction and cDNA synthesis (Method S6), subcellular localization (Method S7), yeast one-hybrid (Y1H) assay (Method S8), and total antioxidant capacity assessment (Method S9), are provided in the Supporting Information.

### Statistical analysis

All statistical analyses were performed using SigmaPlot12.5 software. For pairwise comparisons, Student's *t*-test was applied. For multiple-group comparisons, either Duncan's multiple range test (DMRT) or Dunnett's test was used.

### Accession numbers

The accession numbers of genes used in this study are listed in Table S2.

## Supplementary Material

Web_Material_uhaf291

## Data Availability

The data underlying this article is available in the article and in its online supplementary materials.
