# Supplementary material for: DoMYB75 coordinately regulates polysaccharide and anthocyanin biosynthesis in Dendrobium officinale
Source: Hortic Res. 2025 Oct 25;13(2):uhaf291. doi: 10.1093/hr/uhaf291 (PMC12903456; doi:10.1093/hr/uhaf291)
Supplement: Web_Material_uhaf291 [file web_material_uhaf291.zip › Supporting information.docx]

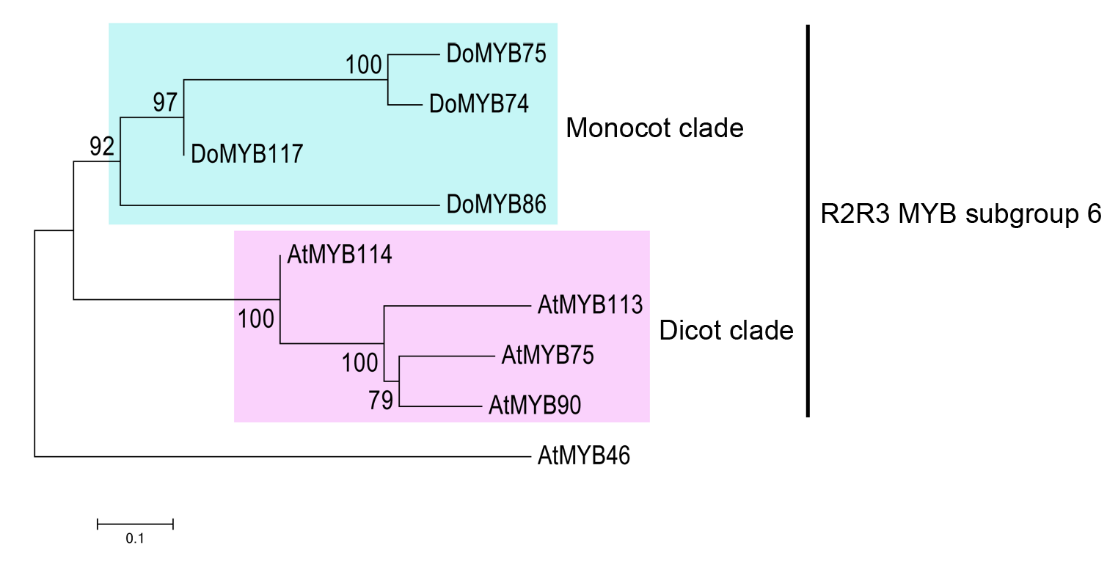


Figure S1 The molecular phylogenetic tree of the R2R3 MYB subgroup 6 proteins from *Arabidopsis thaliana* and *D. officinale*. The tree was constructed using MEGA 7 by the neighbor-joining method with 1000 bootstrap replicates. This construction is based on the alignment of these proteins by ClustalX 2.1.


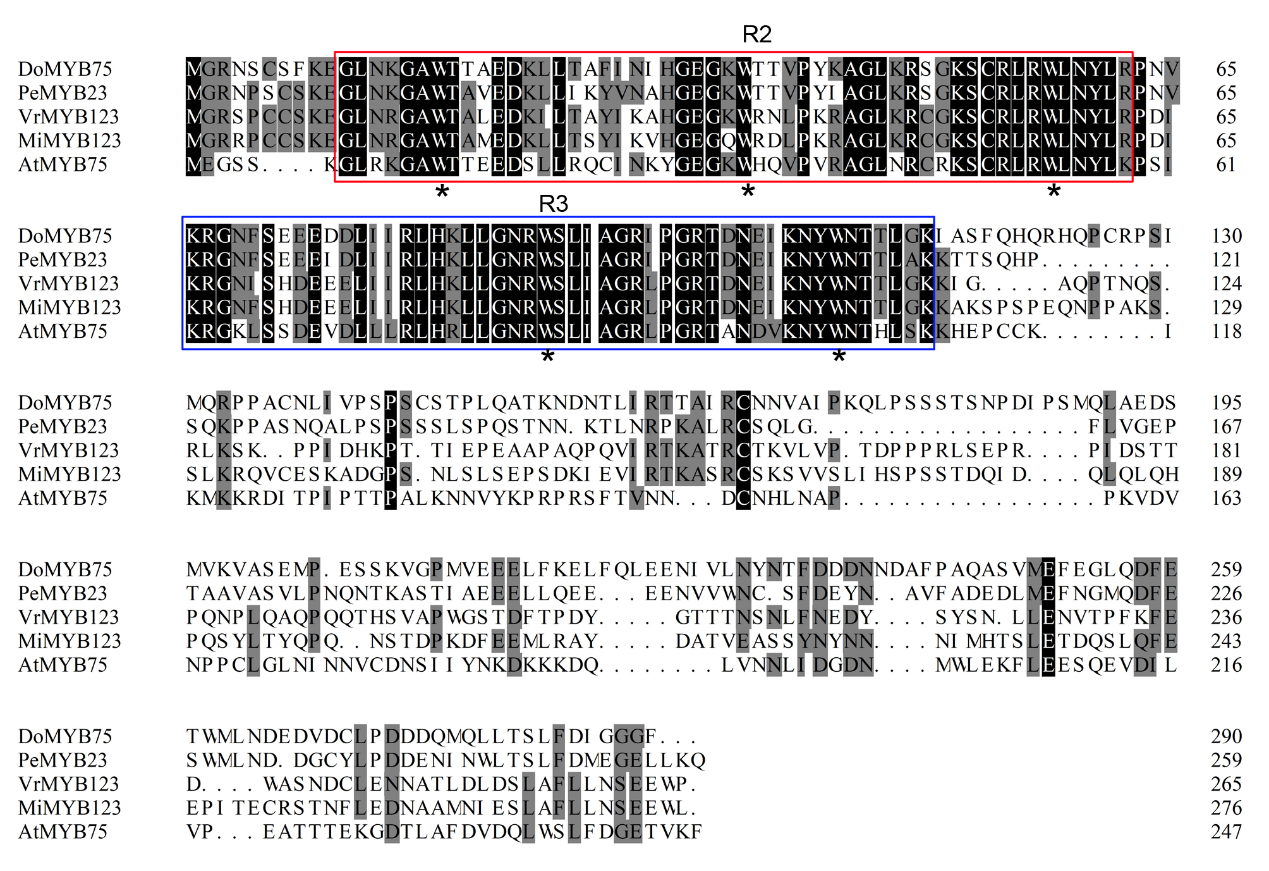


Figure S2 Multiple sequence alignment analysis of DoMYB75 from *D*. *officinale*, PeMYB23 (XP_020582444.1) from *Phalaenopsis equestris*, VrMYB123 (XP_034682583.1) from *Vitis riparia*, MiMYB123 (XP_044472102.1) from *Mangifera indica* and AtMYB75 from *Arabidopsis thaliana* (AT1G56650.1). Multiple alignment analysis was performed using ClustalX 2.1 software. The R2 and R3 MYB repeats are indicated in the boxes. The conserved tryptophan residues (Trp, W) in the R2 and R3 MYB repeats are marked with black asterisks.


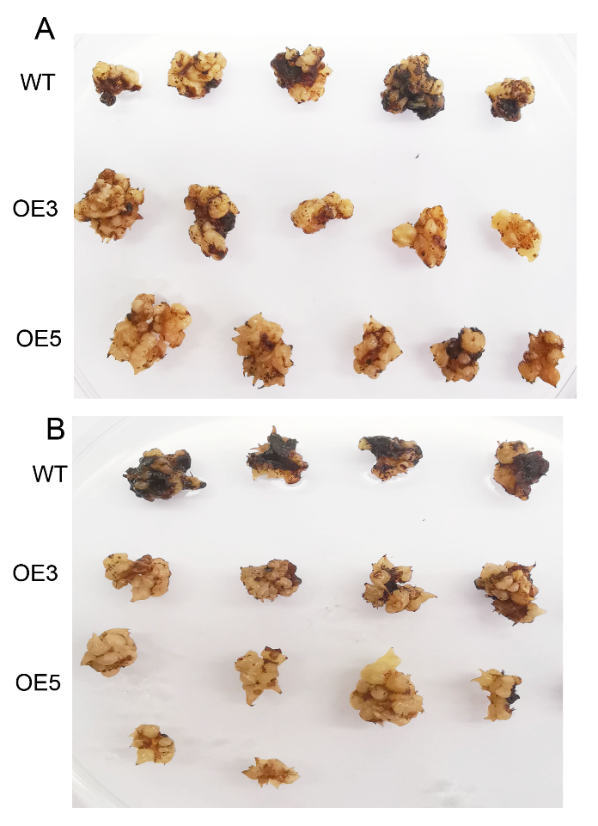


Figure S3 Detection of H_2_O_2_ in WT and transgenic PLBs through DAB staining. (A) Detection of H_2_O_2_ on WT PLBs and *Ubi*:*DoMYB75* transgenic PLBs under control conditions. (B) Detection of H_2_O_2_ on WT PLBs and *Ubi*:*DoMYB75* transgenic PLBs under 15% PEG treatment. The brown area within the images denotes the concentration of H_2_O_2_. WT, wild-type; Two *Ubi*:*DoMYB75* transgenic lines specifically refer to OE3 and OE5.

Supplementary Text S1

The yellow highlighted indicated SMRE.

>ProDoCSLA3

GGCAATATGTGTGAGGTTGATGAGGACAAATTCACTACAAAATCACATAATAAGATTTCATTAGGGTATATATATTTTTTCTTATAAATATACATAATATTATTACACAATTAGTGCATTTTACAAGAATAAAATGAGTTTGTCATTTCTTGAAAATTTACAAAATATTTAATATTTAAAGCATTTTATTTTTAAAATTTTAAGCTATTATATGATCACATAAAAATCCATCTCTATATAAGTTTATTTACTTATGAAAAAAAAAAAACTTATTTATGACCTTTTTTATGTTGAATATTGAAGTATAATGCTTGCACACTTGTTCTAATTTTAATAAATGATGCATGAGAATGGGGCTTAAAAACCAGTAGTGAGATCCACAACAACCTGTTCACGAGGATGAGAAATATCTATTATAATTTTTTCCTATAAAATCATCCATAAAATAAATTTAAATCTTAAGGAAATAAGGCTAATTACAAGCCAATAATATAAGTTGGGTAAGAATTTCAAATATGGAGAGGTATTCTACTATCTCATACAAGCCCATGTATGCAGTGGTCATGGCTTTTACAAACTAATTCATCCCTACCACATAGCACTTCTATCCATGAAAACTCTCCTCTCATGCATAAGAGTTCGACTCTCATTCTTAGAAAATTTTATGCAAATACGTGCCTTACATGCTAGGCCTAAAAAACTCACTCAAATCATATACATAGATCCATCTATACATCATCTTATTACTCCATATGATGTCTTATTTTTTCTATTTATTCTAGATGATGTATTTTATGTAATATTTTTAATCCTATAAATAAGAGAAGAATAGATGTATTTTGAATATTGTGTTATGCGATTTATATAGAGAGATAATAAAACTTTCTAATTACTTTTCATACAATAATTTTTCTCATTTGGTTCTAGCCCTAATCTACCAAAAAAATTATTAAAAATAAACTATTAGTAAAATAAAAAATAATATTAAATTCACCATATTAAATCTATTTTAAAATAAAAGTTAAATAGTTAAAATTTATTTGTATTTAAAAATAAATTATTAAAATATATCACATTTATCACGTTTGAGTTCGATACCAACTTTTTTTTTTTTTTTTTGAGCAAAAAAAAGAACCTCCAACATCGGCTTTTCCCCCGTTAATTAATATAAGCCGCCGTCACGTGCTAGCTGTAAAATTCTGGATGGACAAGGCACCCCGTCGATTCCCTTGCCTTTTAGTTTTTATACCATACAATGCAGAACATCCAACCGGTTTTTAATCGATAGAAGGAAATCCTTGTGGAACAGAAAAGCAGCTGGGAG

>ProDoCSLA9

CCTCTTCGTAAGGCTCGCCCGCCGGAGCCCATCGAAAGTGTACCGCTGGGAGCCATTTGCCGACCAGGTCGACGAGGAGATGCAGCAGGAATCCATCGATAGCTATCCAATGGTCCTCATCCAGATTCCCCTCTTCAACGAGCGACAGGTGGGTGGACCCTTATTACTTTCCATCTCTCTTTCTCTCTCTTTCTCTCAAAACAATGTACTGAGGCAATAATATTATTTATTATTTTGCCACTTATGGCTTGGGCTGGAC AATTAATAAAAGATAAAAAGTAAGTATATTAATTTAATTTAAAATGTTATATCTAATTATGCTCAGGCTTCGGCCACACATTTATATCTGAAAGGGAATAAATAAAGCTATAAATATATTGATGATATTCAGGTTTATCATGTGGCCATTCGTGCGGCGTGCAACTTTTCTTGGCCATCCGATCGTCTTGTCATTCAAGTGCTCGATGATTCAACCGATCTCGTCGTCAGGGTTTGACAGCTAACTTTTTTTTTTTTTTTGCTGAAATATTACTTAATTATGCACGTGACATTATCAAATAAATAAAGTAAAAAAAATGTGGCTGATCAGCTTATTATTATAGAAGGGAAATATATTTATTTTTTGCTAATTTTTTGTCCAATTAAATGCTTAATTAAGAAACTTTGATAACTATTAGATGACCAAATTAATTCAATCATACCGTAAATAATTCTATTAACTTTCTTAATTAGACTATATGCATTTAAGATATTATGTATGGTTGCCAAAATTTTGAGATCAAATTTGGAATATATAAATTTAATGTGTTTAATTAATTTGAAAATAAAAATTTTCATCTCCTTTAAGAAAATAATATAAATATGCTAATTACAAGAGTAGTGTAAGTTCTGAATGAGGCTTCAACTAGTTACAAGTTCCATCAGTACTTAGTAGCAATTTCAGTACGTTAATTGGCCACGTAAATATGCATGAAATATTAATTAATCGTCGTTGCAGAATATTAATTAGTTATCTTTAAATATTAAGGAATCGTAGCTGCATAAAGCATTTTATCAGTCAATTACAGTGCATCGATCATGTTTGTTGTTGCTTTTTGCATGTAAGGAATTGGTGAAGGAGGAATGTGAGAAATGGATAAGAAAGGGGATTAACATAAATCTAGAGACGAGAGATAATAGAAAGGGTTACAAGGCTGGTGCTCTCAAGGATGGGCTGACATGCAGCTATGTACAAAAATGTGAATATTTAGCCATATTTGATGCTGATCATCAACCAGCTCCTGATTATCTCAAACAAGCAATTCCTTTCCTCATACACAACCCTAAAATTGGTCTTGTTCAAGCTCGTTGGAAGTTTGGTAATTCTAAAATTTTGATTAAATTTCTCCTCTAGCTCAATGTGTGTGCGCGCTCCATTTTTATTTCTAAATTCGATTTTGGTTATTGAATGATGATGCTATACAGCCAATGCTGATGAGTGCCTG

>ProDoCSLA12

TCTCTCCAACCTCCATCTCCCAACTACAGTGATCCTACCAACCAAAATATATAACATGCCAACATTCTCTTGAGAATAGCTCTCAAATCATTATATTCTTCCAACCCCAACTTTGTAATGTGATTTATATTTTTTATATTAAGCCAACGTTTTCCCCCCATGAAGGGGATCATTTGTAATCAACAATACACACTAGTGGATTCATTTCAACTGGACAAGACCTCGTGGTTTTTCTCCCTTTTCAAGATTTCCCATGTTAAAATTATGTTGTTTCTTAAGACACTTGCACCCCTCTCTTTTGATCTGTATGTATGCTAAATCTTCGTTCAAAACTAAGATGCTAAGCACTTTATCTATTTACCGTCGTAAATATTCCAGAACGCAACAAATAGTGTTGTGCTTCACCTCGACCTATAAAAAGAGGTCATAGGGTAAATTCATGGCATATGGTATCATAGAGTCTCTCCCTAAGCTCTCTCTCTCTTTCTCCTTCTATGTTATTTAATCTTTCTTATCTGTATAATTTTACTCATGAATAAATTTGAAACCAACACACCAATAATAATTGAGATAATCAGTGAGTTCCCTAAAGGTTGGAGTTGGTCACTCAACCTAACCTTAAATAATGATTCATGTTGTATTCGGTCCTTTCAAGGAAAAAAAAAAAACATTATTGAGATATTATTTTTGGTCGGTCGATAATAAAACAATCTATCAGTACGTATTTTGAAACGGCAGCATTGCTTTAGCTCACAGCTCAAACCATTAAACATCAATCACATACAAAAGTATAGAACGGTCCAGCTACACCTTCGAATCAAGATGAACTCCCTTTCTTGGTGCATTATTAGAGGATCCTATGTAGGAAAAACTATATTCTCTCTCTCTCTCTCTCTCTCTCTCTCTCTCTCTCTCTCTCTCTCTCTCTCTCTCTCTCTCTCTCTCTCTCTCTCTAAATTTATAGAATAAAAATATTCATGGACTATCAGTAATGTTTAAGCTTGTTTCAGATGCAGACATTTCAACGTTCATTTCTTCTTTTATAAAGAGTTTCATTCCTTCAACAAAGCAATAATTTCCATCGATCATTACAAGAAAACAATAATTTCTCAATCATAATTCTATTATTTGAAGAACAAGCTTCAATAATAAACACAACTGCAAGGTCTATACTCCCTCATGTGTTCTAATTTCTATATGAGCAAACCCATCTGCTACATCCATGTCTACAAATATTCAGCATTAACATTAGCAAGGCAGGAGAAAGAAGAATATGGAAGGTTAAATTAGTTTAAGTTCCTCAGCACATTCTATATAATTATTTCCTTTGTTATGATTATACACTAATTAGCAATAATTTTGTTTTTCTATCTCAGGTGTAATTACATTTTCGCTCTATGAGCATATTCGTATGCTGATTGACAGGTTGTTATATCACATATGGATGACACTATTATGGATGAGGGTGGTTGTGATTTTACCGATAGCGAGAAAGATTATAGTTGCATTAATGTGGACGAGGAGGGTCTGATTTTACCGATAGCGAGAAAGATTATAGTTGCATTAATGTGGACGAGGAGGGTC

>ProDoCHS

CCTTTGTCGCCAAACAAAGGTCTTTATGACCTCCGTCGCTTACCGATTATGGTTCTTCCGACTAAACGAATACATAAACAATGACTCTTTATATTCCCACCATACAATGTACTCCATATGATGTACATCAAGCCAAGGCCTCCCCCCCGGGAAGGGCCTTATTTGTAATTGACTGCAAATAACAGTGGATGCATTCAAACTGGGCCATGTCCCGTGGTTTTTTCCCTTTTAAGAGTTTTCCACATAAAAAATATTAATGTCTCTTATTAATACCACACCCTCTCTCTTATCTGATTGCGTGTTGGATGTTCATATAAAACTAAGACGTTAATCTATATATTACTCTACCATCACGAATACTCCAGGATGCAACAATAAGAATAAACATATATTTAAGAAGATTAATGTATCAATATAGTGATTTCAAGATTGAAAGACTGCAATAATTTTTTTATTCAATCTTCAAGACACGTTCATGATTGATCAAATGATCAAAAAAGCTCAAGAGAATATTTTAGAGTCATGAAACACTGAATTGTAATTATAACATGATTGACTCCTATTATTTGCTCAAGCTTTTAGTTATAAAATTCATTAAAATTATTTTGAAAGCAATATATTTAAAACTTAGCAAAATTTACAAATTAAATCCATAATTTTCTTAGTTTTTTATCAAATATATTTTTAATTAAAATCCATAATTAATTATACAACTGCAAAATTAACTAATAATTGAAAATTTTAAATGCAAACCCAAGTTTTAATTAAAAGTTCTATTCAAATTTAGTAAATTATTTAGAAAGAAAAGAGGTTAAGAAAATATTGCCTCATAGAATAAAGGAGTTAATTATCACAATTAATAAGAGGAACATATTTTATTAAAAATTTAAGAAAGAACTAATTCATCAATATAATTGACGCATAATGTATCAAAAAAATTGACACATTCTTAACAAGCTAAATGTTTTGGCGTGTTGTTCATTAAAAGAGAGAGAGTGACAGAGAAAGAGAAAGAGAAAGAGGTGGAGAGGAATGAGAGTGAGAGAGAGAGAGAGAGAGAGGAGAGGCAGCCGACACTTTAAACCAGCGAGCAATTTTTAATTTTTTATTCTAATTATTTAATTTAATTTTTTTTATAATATTTACATAATTTAATTAATATTATGTTTATATTATATTTATTTTAAAATAAATATAATAGTTATAAAAATTTTAATATTATAGTTGTAAATATTTAAATGATTTTTTTAATAAATATTAATTAAATTTATAGTTATAAATATTAATTAAATTTTTTATAAATATTATAGTTTATAAATATTAATTAAATTTAAAATTTATAAATATTAATTAATTTTTTTATAACTATAAGATTTAAATTTTTATAACTATTATATTTATTTTGGAATAAATAAAATATAAAAATAATATTAATTAAATTATGTAAATATTATAAAGAATTAAAAACCCTCCCTGGTTTAAACTGCCCGCTGCCGCTCCTCTCTCTCTCTCTCTCTCATGCCTCTTCACCTCTTTCTCTTTCTCTGTCTCTCTCCCTTAATGAACATTCCAAAACATTTAGCTTTTTAAGAACGTGCCAAATTTTTTGATGCATTATGAATGTATCAATTATCTCATTTTTAAATTTCTAAAGGAGAAAGCTCCTCTCATTAATTGTGAATATGATTAACTTGATCCTTTATAAAATTGTTCCTTCTTTGCTCACATTCTATCACTTGATCGGTTCTAGCAGCTAAGGACTGACTTGTCCCACCTTCCTCTAAAGCAAAACACAAAACTTTTCACC

>ProDoCHI

TGTGCCATCAGAATATGACATTTTTTTCTAATTAATGTATTATGCAGCTACTCACGATACGTCATCTGTACAGATTGATGATCTAGTACTCAGCTAATCATCAATATCATTCATTTATTTATGATGTACTAATATCAGCACGATATGGGCGAACGAAGTACTTACAATCTGTAGTATTAATTGATTGTATTTAGCTACCAATAATCTTACTATTATTTATTACTAACTATCTTAGCATGCAAAAATATATTTATATGTTAGATAAAGTAAGTGAGTGAATAGCTGTCTTTCAGTGAGCAAACATTTATCTGTTGTCTACTGCAGTAAATTAGTAAGTAATTTTTATAAAAATAATAAGAATAAATTAGTGGTTAAAAGCTCTTTGAAGCTAAGCCTAAATTATAAATAATCAATACAATAAAGAAACTGTTAATTGTGGAATTGTTTGAGACAATTTTTTTACTAATTTTTTAGTACAAAATTTTTTGAACTTAAAATTATTATATTAAAAAGTTAAATAAGAAAGTTATAAAAAACTGTCTAAAAACATTAATCTTTTGAAAAATATTAGAATTTATGAGCAAAAAATTTTCAATGCTTTAGTATTAAAAGCACAAAAGAAAAAAACATTGATGCAAATATAAATGAAGATAATTGTTTAATTATTTTTAGACAAATTTTCGATTTGTCTCTGTCAACAATAAAAACGTAATTTCTGTTCACCAATACATTGGTACTGTTTACAAAGATAAATCTGAACGATTCAATGTATTCAATCTCTGCACTTAATAATCTCTACTATTTCTAAAGTTTCTTTCTAGTACAATTAAAAAAATAATGAAAAATTAATGACTAAAATCTTTTCCAAAAGCCTAAATTATAAATAAATGAAGCTATCAACATTTATTGATAGCATTCAATCGCCTAATGACAATAAAGCATCTGTTAATGTTGCCTTGATGGAATCTCTGTTTACAGCTCATATGGTAACCTGCTATTAAAGAAATAAATTTTAAAAATAATATATTTTTCTTTCATCCGTTGAGCGGTCGCGCGACGGATCACCGACTCTCCGGATATTTTCCTTAAAAATTCTCAAATAATCGATCGGGCGGTTAGGTTCCTTCGAGAAGCTGACGAGGGTGACG

>ProDoANS

GAAATTTGAGCAACACTCATCCAAACAAAAAATTTTACAAAAAATTGATCAACCTATACAAAGAAGAATGTAGGAGAAGTTAAAAATATAATTCTCAACCAAATTCATCCAAAGTCACCTAACTACAGTCATTTAACCAAATCAAGGGCTAAAATTATAATGAAGATTCAAAATTTTAAACAACTCAAATAGTCAAAGCCCCTAAGAAAACCAATCAAAGAAGTTTAGTCTGAATACTATAACTTACAGCATTTAAAAGAATACTCAACTCAAAAATTCTTTCTATTAAAGGATGAGATTGAACCTTTGATTCACTAAGGATATTTAAGTCATTATATATAAACATGACTTATAGAGCATCAACATCATGAATAAGGTCCTCACTAGAATGACCCTATGAGGAAAGTGGCAGACAAAAGAAATTATTGACATTATTGAATAAGAGTACTATAATATTTAATGTTATTTAAAGCATCCAACTCTTTAGATTTTTAAAATTATATATCTTTTAAGCATGTTTTCTTAAAAATATTAATGAAAATTAATATAAATAATATTGTATATAATAATAATCCTAGGAAGGCATGCCCCTGAGGAAGCCTTGAAAGGAAGCTAATAGTGAATAAGGTCCTCACTAGAATGACCCTATGAGGAAAGTGGCAGACAAGGTCTCGACTCACGAACCCAGAAATGCATGCCCATGAGGTATCCACGAAGGAAATTTAGTAGTGAAGAAGGTCCTCATTAGAAAACACCACATGAGGATATGTGGTGTCTATGGTCTCAACTCACAACCCCAAGACGGCATGCTCATAAGGTAGCCTCGAAGAGAAGCAAGTAGTGGATAATATCCTCACTAGAAATACTCTATGAGAGAAATTTGTTGATAGCGTCACCACTCATTCAAAAGCCTCAAAAGTTAAAAACTACATAAGGTTTAGAATTATTTAAAGACCTTAGAATATATATTAATTATTGTTAAGAAAAAAATAAAAAAGTTAAGAAAATAAAAATTGGGGCAGTGTAGAACTGTAGATAAAAAAAAGTAAAATTAATTTATTTTTATTTTTTGGAACGTAATCATTATTCTCAATAAAATCTAATAATATTTTATATTTAAATAATATTTAAGAAATCTAATATTATTTAATTGATAATAGTTTTTTATTAAAATATTTAAAAAATATAATATTTTATTTTTAAATTAAATAATACTTTTTATTGAAACATAATCAATTAATTAATATAGAAAATTGTTTTAAATATTAAATTATAAATTAATGAAACATTAAATTAAAAATTAATGATATTTTAAATTAAAAGTGAGACAAATTTAATTATAAAAACTTTAACTAAAAACTTATAGTTAAAATAATAAATTAAAAAAGTTATAATTGAAATGTAAAATTAAATTATTTTACTTTTTTGTATCTACACTGCGTCGTTTTTTATTTATTTTTTTCTTATATATATATATATATATATATATATATATATATATATATATATATATATATTAAGAAACCAGATATAATATTAAACAGAAAAAAAGTTATTAAGAAAACGGATATAATATTAAAGCGGAAAATAACATTTTATTAATAAACCGGATATAATATTAAACCGGAATTTTTTTTTTCTTAAATTCAAAAAGTAGAACGGAATACCGATTTCTATTGAAATCAGATAATGAAGTTTCCATTTCTTTTTAAACCGGATAATTAAAAAAAAAAAATATTTGCTCTTTTTTTTAAAAAAAATTGTTCGTTGATATCCTCCTTAAACTAAACCTTAAACTAATTTAAACTTAAAATCTAATCATTTTTATAAATTGGCCCCAACTAACCATACAACTAAATTTATTTGATGACCGACAACAGCACTCGTTTAATAAAGAGTAATGTCCTGAAAATAAAAAAGAAAATTTATATTTCATATTTATCTTTGACATATAGAAAATTTGTGTGTTTTAAACATTTACTCGCTTCCTATTATTCATTATTACAATTTTTTTATCCCCATTTATTTTTCTTCTTTTATGCGTGGGTGGGCCGATAGTTAGGCATGTGCTTTGCTAACTTCCTTTCTTCACCTTCCGGTCTTTCCCTAGCTACCTATCAATTCCATTTCCCCACTTCTCCACAAGCAGACTCCGCACCATAAAATCCTCGCCATTCTTCTTCCTTCCCAGCCAAAGCTACTCACTAAGAATACACA

**Method S1**

**Vector construction and Arabidopsis plant transformation**

The promoter of *DoMYB75* (*proDoMYB75*) was isolated and cloned into the pCABIA1391Z vector to generate a *proDoMYB75:GUS* construct. This construct was transformed into Arabidopsis plants by *Agrobacterium*-mediated method. The primers used to construct the *proDoMYB75:GUS* vector were listed in Supplemental Table S1.

**Method S2**

***β*-Glucuronidase (GUS) staining**

Arabidopsis transgenic seeds were surface sterilized with 1% sodium hypochlorite and sown on 1/2 MS medium. After stratification in darkness at 4 °C for 3 d, the seeds were cultured in a growth chamber under the condition 16-h photoperiod (100 µmol m^-2^ s^-1^) at 22 °C. Seven-day-old seedlings were carefully transplanted to the 1/2 MS medium containing 15% PEG6000 or 150 mM NaCl for stress treatments. The seven-day-old seedlings grown on the 1/2 MS medium were subjected to low temperature (4 °C) for cold stress treatment. After 24 h, the seedlings of each treatment were collected and used for GUS activity analysis. The Arabidopsis plants were grown in soil (topsoil and vermiculite; 1:2) under 16-h photoperiod (100 µmol m^-2^ s^-1^) at 22 °C. Roots, stems, flowers and leaves from the *proDoMYB75:GUS* transgenic Arabidopsis lines were harvested and used for GUS activity analysis. The plant samples were incubated with GUS staining solution in the dark at 37 °C for 12 h, then decolorized with 70% ethanol to remove chlorophyll. The stained samples were photographed with a Leica DVM6 video microscope (Leica Microsystems Ltd., Heerbrugg, Switzerland).

**Method S3**

**Construction of overexpression vector and transformation of *D. officinale* orchid**

The full-length coding sequence of *DoMYB75* was amplified and cloned into multiple cloning sites (MCS) of the pOx vector, which contains a maize *ubiquitin* (*Ubi*) promoter before MCS. The recombinant construct was confirmed *via* sequencing analysis at Tsingke Biotechnology Co., Ltd., and then the confirmed construct was introduced into *Agrobacterium tumefaciens* EHA105 strain. Transformation of *Dendrobium* orchid was performed as described previously (Zeng et al. 2023). The primers used to construct overexpression vector were listed in Supplemental Table S1.

**Method S4**

**Anthocyanin analysis**

Anthocyanin analysis was conducted as described previously(Li et al. 2020). Briefly, samples (100 mg) were crushed into fine powder in liquid nitrogen using a mortar and pestle, and extracted with 2 mL 1% acidic methanol (containing 1% HCl). The mixture solution was extracted in darkness at 4 °C for 24 h, and then centrifuged at 12,000 rpm for 5 min. The supernatant was transferred to a fresh tube, and then measured the absorbance at 530 and 657 nm using UV-6000 spectrophotometer (Shanghai Metash, Shanghai, China). The relative anthocyanin content was calculated as (OD_530_ ‒ OD_657_) ÷ FW (fresh weight).

**Method S5**

**Determination of monosaccharide content**

PLB samples were dried and ground into fine powder. The powder samples were pre-extracted twice with 80% (v/v) ethanol to removed monosaccharide, disaccharide and oligosaccharide. Water-soluble polysaccharides (WSPs) were extracted with distilled water at 100 °C for 2 h. The WSPs were hydrolyzed to monosaccharides with hydrochloric acid, and then the hydrolyzed sugars were used for derivatization with 1-phenyl-3-methyl-5-pyrazolone (PMP). The monosaccharide content was analyzed by HPLC under the following conditions as described previously(He et al. 2015).

**Method S6**

**RNA extraction and cDNA generation**

Total RNA of *D*. *officinale* samples was isolated using a RNA extraction kit (RNAout 2.0, Tiandz Inc., Beijing, China). The mRNA was reverse transcribed into cDNA using the GoScript™ Reverse Transcription System (Promega, Madison, WI, USA) according to the manufacturer’s protocol.

**Method S7**

**Subcellular localization**

The full-length coding sequence of *DoMYB75* without termination codon was cloned into pSAT6-EYFP-N1 expression vector to generated a *DoMYB75*-*YFP* (yellow fluorescent protein gene) fusion construct. The fusion construct was transformed into protoplasts of Arabidopsis mesophyll by PEG-calcium transfection method(Yoo et al. 2007). Transformed protoplasts were incubated overnight at 22 °C in the dark, and the YFP fluorescence signals were detected by Leica TCS SP8 STED 3 × microscope (Wetzlar, Hesse, Germany). The empty vector pSAT6-EYFP-N1was used as the positive control. The primers used for subcellular localization were listed in Supplemental Table S1.

**Method S8**

**Yeast one-hybrid (Y1H) assay**

The promoter fragments of *DoCSLAs* and *DoANS* were amplified and inserted into the pHIS2 vector to generate pHIS2-*ProDoCSLA* and pHIS2-*ProDoANS* constructs. The full-length coding sequence of *DoMYB75* was cloned into the pGADT7 vector (Clontech) to generate pGADT7-DoMYB75 effector vector. The pHIS2-promoter and effector vector was transformed into the yeast Y187 strain using PEG/LiAc method. The transformed yeast cells were cultured in SD/-Trp/-Leu (SD/-T/-L) medium for 2 d at 29 °C. Then the positive colonies were confirmed by polymerase chain reaction (PCR) and placed onto SD/-Trp/-Leu/-His (SD/-T/-L/-H) medium containing 3-amino-1,2,4-triazole (3-AT). The yeast cells containing pHIS2-promoter and pGADT7 were used as negative control. The primers used for Y1H assay were listed in Supplemental Table S1.

**Method S9**

**Total** **antioxidant activity analysis**

Plant samples (0.1 g) were extracted with 1mL extracted buffer, and the homogenate was centrifuged at 10000 rpm for 10 min at 4 °C. Then the supernatants were collected and used for antioxidant activity analysis. Antioxidant activity was measured using a T-AOC kit (Solarbio life science, Beijing, China) according to the manufacturer’s instructions. At least three independent replicates were performed.

References

He C, Zhang J, Liu X, Zeng S, Wu K, Yu Z, Wang X, Teixeira da Silva JA, Lin Z, Duan J. Identification of genes involved in biosynthesis of mannan polysaccharides in *Dendrobium officinale* by RNA-seq analysis. Plant Molecular Biology. 2015; 88: 219-31.

Li Y, Shan X, Tong L, Wei C, Lu K, Li S, Kimani S, Wang S, Wang L, Gao X. The Conserved and Particular Roles of the R2R3-MYB Regulator FhPAP1 from *Freesia hybrida* in Flower Anthocyanin Biosynthesis. Plant Cell Physiol. 2020; 61: 1365-80.

Yoo S-D, Cho Y-H, Sheen J. *Arabidopsis* mesophyll protoplasts: a versatile cell system for transient gene expression analysis. Nat Protoc. 2007; 2: 1565-72.

Zeng D, Si C, Zhang M, Duan J, He C. ERF5 enhances protocorm-like body regeneration via enhancement of *STM* expression in *Dendrobium* orchid. Journal of Integrative Plant Biology. 2023; 65: 2071-85.
